# Multidimensional exposure architecture shapes vaping-associated transcriptomic dysregulation in oral epithelium

**DOI:** 10.3389/fonc.2026.1838256

**Published:** 2026-06-01

**Authors:** Jessica George, Stella Tommasi, Niccolo Pabustan, Daria M. Kessler, Zairah L. Thomas, Lourdes Baezconde-Garbanati, Kimberly D. Siegmund, Ahmad Besaratinia

**Affiliations:** 1 Department of Population and Public Health Sciences, USC Keck School of Medicine, University of Southern California, Los Angeles, CA, United States; 2 Norris Comprehensive Cancer Center, USC Keck School of Medicine, University of Southern California, Los Angeles, CA, United States

**Keywords:** differential gene expression, dose, electronic cigarettes, exposure, RNA sequencing, smoking, transcriptome

## Abstract

**Introduction:**

Electronic cigarette (e-cig) use (vaping) has been associated with dysregulation of genes and molecular pathways in epithelial tissues. However, the relative contributions of dose and product characteristics to vaping-associated transcriptomic alterations have not been systematically evaluated.

**Methods:**

We performed RNA-sequencing of oral epithelial cells from e-cig users (vapers), cigarette smokers, and non-users. Differential gene expression was assessed using covariate-adjusted limma-voom modeling with false discovery rate control. We evaluated the extent to which exposure-specific dose metrics (including cumulative e-liquid, cumulative e-nicotine, years vaped, and plasma cotinine for vaping, and pack-years and plasma cotinine for smoking) explained transcriptional changes.

**Results:**

Among vapers, we additionally examined whether device generation and flavor type contributed to variation in gene expression. Both vaping and smoking were associated with transcriptomic dysregulation relative to non-users, with partial overlap in differentially expressed genes (DEGs). Functional enrichment analyses revealed disruption of shared cancer- and signaling pathways, including RHO GTPase Cycle, as well as perturbation of pathways specific to vapers or smokers. Among vapers, 27.6% of DEGs showed concordant behavior across all dose metrics, indicating heterogeneous dose-response patterns for the remaining DEGs. Device generation and flavor type explained additional, largely non-overlapping components of gene expression variability. A much higher proportion of smoking-associated DEGs (54.1%) was consistently affected across dose metrics, reflecting more unified dose-dependent responses.

**Discussion:**

These findings suggest that vaping-associated transcriptional dysregulation reflects combined influences of dose and product characteristics, highlighting structural differences in molecular perturbations between vaping and smoking. Incorporating multidimensional exposure metrics and product features into regulatory evaluation may better capture the biological complexity of e-cig exposure, thus informing clinical, public health practice, and regulatory decisions.

## 1 Introduction

Electronic cigarettes (e-cigs) are handheld battery-powered devices that simulate smoking by aerosolizing a special liquid (e-liquid/e-juice) for inhalation (1). Although nicotine-free options are available, the e-liquid is typically composed of varying concentrations of nicotine, propylene glycol, glycerin or vegetable glycerin, and flavorings (1, 2). Since their introduction to the US market about two decades ago, e-cigs have rapidly evolved from the first-generation ‘cigalike’ devices into second- through fourth-generation systems with sophisticated designs capable of delivering higher nicotine doses and improved user experience (1). Concurrently, the emergence of thousands of flavored e-liquids with sweet, fruit, and mint profiles has increased product appeal and contributed to the growing diversity of e-cig products on the market. The use of e-cigs is often referred to as ‘vaping’ and e-cig users are commonly called ‘vapers’ (1, 3). E-cigs are widely promoted and marketed as a harm-reduction alternative to combustible tobacco cigarettes and as a tool for smoking cessation (4). The Centers for Disease Control and Prevention’s 2022 National Health Interview Survey Highlights reported significant changes in current tobacco product use from 2019 to 2022, showing increased current e-cig use and decreased current cigarette smoking among nearly 15 million adults (5). Importantly, adolescents and youth comprise a major segment of the vaping population (6, 7). The ongoing debate over the safety of e-cig use, its efficacy as a putative strategy to help smokers quit, its potential as a gateway to combustible cigarette smoking, especially among tobacco naïve youth, and its largely unknown long-term health effects make vaping a major public health concern (8, 9).

E-cig aerosol contains many harmful and potentially harmful constituents (HPHCs) as those found in tobacco smoke, although in fewer numbers and at substantially lower concentrations (1, 10, 11). These include carbonyl compounds, volatile organic compounds, free radicals, and heavy metals, among others (3, 9). While the reduced number and lower levels of HPHCs in e-cig aerosol align with harm-reduction principles, they do not equate to absence of health risks (6). Many HPHCs are known to exert their biological effects through transcriptional dysregulation of genes involved in the pathogenesis of diseases, like cardiovascular-, immune-related (inflammatory), and respiratory diseases, and cancer (1, 9, 12). Thus, examining transcriptional regulation in cells and tissues of vapers can help determine the disease-causing potential of vaping.

The oral epithelium is the first anatomical site directly exposed to the chemical constituents of e-cig aerosol and tobacco smoke (13, 14). It serves as a sensitive interface where the biological effects of e-cig aerosol or tobacco smoke can be investigated. We previously used RNA-sequencing (RNA-seq) to profile the oral transcriptome in healthy adult vapers and cigarette smokers as compared to non-users (non-vapers nonsmokers). We identified significant dysregulation of functionally important genes in the oral epithelium of vapers, with expression patterns that were partly similar to and partly distinct from those observed in smokers (15). Consistent with our results, other transcriptomic studies in multiple epithelial tissues, including bronchial, nasal, and oral cells, have demonstrated that vaping alters expression of genes involved in inflammation, immune response, and cellular stress (16–21). In addition to transcriptomic alterations (22, 23), e-cig use has been associated with epigenetic changes linked to disease risk. Studies have shown that vapers exhibit aberrant DNA methylation and hydroxymethylation (24–30) and dysregulation of noncoding RNAs (31–34), which are implicated in the initiation and progression of a wide variety of diseases (35–41). This is consistent with the growing evidence on the association between e-cig use and disease outcomes (8, 42–48). Building on these findings, the present study investigates, for the first time, the impact of usage rate (*i.e.*, intensity and duration of vaping = dose) and product characteristics on gene regulation in the oral epithelial cells of healthy adult vapers. In addition to vapers, two counterpart groups of cigarette smokers and non-users have been used as comparators to contextualize the results. The present study is distinct from our previous study (15) in that it extends group-level comparisons to a thorough examination of the influence of dose and product characteristics on gene regulation. Our study hypothesis is that vaping- and smoking-associated dysregulation of genes in the oral epithelium is dependent on the intensity and duration of tobacco product use (dose) and/or tobacco product characteristics.

We first performed RNA-seq analysis on oral epithelial cells of vapers, smokers, and non-users. Subsequently, we applied the limma-voom framework with quality weights to identify differentially expressed genes (DEGs) in vapers and smokers as compared to non-users, while adjusting for age and sex (49–52). Sensitivity analyses were then performed to evaluate the contribution of vaping dose, smoking dose, and vaping product characteristics to changes in the expression of the identified DEGs. More specifically, we conducted ordinal sensitivity analyses to determine the impact of vaping and smoking dose on gene expression levels using exposure-specific dose metrics, including cumulative e-liquid (in milliliters), cumulative e-nicotine (in milligrams), and years vaped for vapers, and pack-years for smokers. Plasma cotinine concentrations were used as an additional exposure dose metric for both vapers and smokers. Moreover, we conducted nominal sensitivity analyses to examine the influence of product characteristics, including device generation and e-liquid flavor, on gene expression levels in vapers. Finally, we performed gene ontology (GO) and functional analyses to identify the biological processes, molecular functions, cellular components, and diseases associated with the DEGs detected in vapers and smokers.

## 2 Materials and methods

### 2.1 Ethics declarations

All research was conducted in accordance with the Declaration of Helsinki of 1975 (https://www.wma.net/what-we-do/medical-ethics/declaration-of-helsinki/). This study was approved by the Health Sciences Institutional Review Board of the University of Southern California (HSIRB-USC), under the protocol number HS-16-00175. Written informed consent was obtained from participants prior to enrollment in the study.

### 2.2 Study population

Healthy male or female adults of diverse ages, races, and ethnicities who could read and write in English were considered eligible for the study. Full details on subject recruitment and enrollment, and inclusion and exclusion criteria have been previously described (15). Briefly, participants were recruited from the Greater Los Angeles Area through online advertisements (Craigslist, Reddit, and myUSC [ https://my.usc.edu]) , social media (Twitter, Instagram, and Facebook), and flyers distributed at local colleges, universities, and vape shops. Initial eligibility was assessed through an online survey and follow-up phone screening, with final eligibility confirmed during an in-person interview. A structured questionnaire was administered during the interview to capture information on demographics, socio-economic status, occupational and residential history, family history of disease, and use frequency and patterns of e-cigs, cigarettes, or other tobacco products, dietary habits, lifestyle, alcohol, and prescription- or over-the-counter medicine (*e.g.*, vitamins and multivitamins). Clinical staff performed oral- and general health assessments of all participants. Those with known respiratory diseases (*e.g.*, asthma or chronic obstructive pulmonary disease), immune system disorders, diabetes, kidney diseases, body mass index of <18 or >40 kg/m^2^, local or systemic inflammation or infection, or any medical disorder or medication affecting their safety or study results were excluded. The following criteria were also exclusionary if present within the past 12 months: any unstable or significant medical condition, pregnancy, uncontrolled mental illness, or substance abuse or inpatient treatment for those conditions. Other exclusion criteria included the use of recreational or illicit drugs (*e.g.*, marijuana or heroin) or any medication known to induce or inhibit the CYP450 2A6 enzyme in the past six months. At the end of the interview and health assessment, those who met all eligibility criteria were provided with a detailed explanation of the study and its procedures and the opportunity to ask any questions they might have. Individuals who agreed to participate in the study, were required to provide written informed consent.

The study population was comprised of 83 subjects divided into three groups, including (I) current vapers (n = 35), (II) current smokers (n = 24), and (III) non-users (n = 24). For subject classification, we used the following criteria: vapers were those who reported current use of e-cigs for at least three times per week for a minimum of six months, and no use of combustible cigarettes or any other tobacco products in the past six months. Smokers were those who reported current smoking of tobacco cigarettes at least three times per week for a minimum of one year, and no use of any other tobacco products, including e-cigs, in the past six months. Non-users were those who reported no use of any tobacco product (e-cigs or combustible) more than five times in their life, with no use in the past six months. Given that e-cigs were a relatively new product at the time of enrollment, we defined the minimum use durations of six months for vapers and one year for smokers to ensure sufficient group representation.

### 2.3 Sampling and processing of oral epithelial cells

Subjects were required to abstain from eating, vaping, and smoking for at least one hour before sample collection. After a vigorous mouth rinse with water to remove saliva, residual food particles, and mucosal debris, oral epithelial cells were obtained by brushing the inner surface of each cheek in a rotary motion using an Ultra Soft Oral-B brush (SENSI.SOFT™; Cincinnati, OH, USA). The proximal, central, and distal regions of each cheek were brushed 15 times, and harvested cells were dislodged into two tubes (one for each cheek) pre-filled with 35 mL of ice-cold sterile phosphate buffer saline (PBS). The tubes were centrifuged at 800× g for 5 min at 4 °C, cells were re-suspended in PBS and pooled into a single tube, and re-centrifuged as described. The collected cell pellet was snap frozen and preserved at -80 °C until further analysis. All samples were processed in a blinded fashion to minimize bias. We have previously confirmed the reproducibility of this protocol to collect several million cells, the majority of which being intermediate and suprabasal oral epithelial cells (15).

### 2.4 Sampling and processing of peripheral blood

Peripheral blood (30 mL) was drawn by venipuncture. Plasma was collected by centrifugation and aliquoted into multiple microtubes (53). All samples were snap frozen and preserved at -80 °C until further analysis.

### 2.5 Quantification of plasma cotinine by ELISA

Cotinine is a prominent metabolite of nicotine (54). Plasma concentrations of cotinine were measured using a solid-phase competitive enzyme-linked immunosorbent assay (ELISA) kit (Abnova Corp., Walnut, CA). Aliquots of plasma samples and standard controls were loaded in triplicate (10 µL each) onto a 96-microwell plate pre-coated with polyclonal antibody raised against cotinine. Cotinine horseradish perioxidase enzyme (100 µL per well) was added, and the plate was incubated for one hour at room temperature in the dark. Wells were then washed six times with distilled water (300 µL per wash) to remove unbounded material. A chromogenic substrate (3,3’,5,5’-Tetramethylbenzidine; 100 µL per well) was added, followed by a 30 minute incubation at room temperature. The reaction was stopped with 100 µL stop solution per well, and absorbance was measured at 450 nm using an iMark™ Microplate Absorbance Reader (Bio-Rad Laboratories, Inc.). The assay sensitivity, defined as the minimum cotinine concentration producing a three-standard deviation signal above background, was 1 pg/µL, with a detection limit of 5 pg/µL. Samples with undetectable cotinine were assigned one half of the detection limit (*i.e.*, 2.5 pg/µL).

### 2.6 RNA-sequencing analysis

Total RNA was extracted from snap frozen oral epithelial cells using the RNeasy Mini Kit (Qiagen, Valencia, CA, USA). RNA quality was assessed in an Agilent 2100 Bioanalyzer using the RNA 6000 Nano Chip kit (Agilent Technologies, Santa Clara, CA, USA). RNA-seq libraries were prepared from 300 ng of total RNA samples using the Kapa HyperPrep kit with RiboErase (Kapa Biosystems, Wilmington, DE, USA). The protocol involves rRNA depletion, cDNA generation, end repair, A-tailing, and adaptor ligation by polymerase chain reaction (PCR). Samples were multiplexed with unique adaptors and sequenced on an Illumina NextSeq 500 platform (75 base pair single-end reads). Data quality was evaluated with Illumina SAV, and demuliplexing was performed using Illumina Bcl2fastq2 v2.17. To minimize technical bias, library preparation and sequencing for samples from different groups (vapers, smokers, and non-users) were performed randomly and in a blinded manner.

RNA-seq data were trimmed, aligned, and quantified using Partek Flow (Partek Inc., St. Louis, MO). Sequenced reads were trimmed from both ends based on quality (Phred ≥ 20 and minimum read length = 25 nt). Trimmed reads were aligned to the human hg38 reference genome using STAR with default parameter settings (51). Gene and transcript abundances were estimated from aligned reads with Partek E/M method (50). All 83 samples included in the analysis had sequencing depth of >15 million reads/sample and >50% read alignment. Post-alignment processing, quantification, and differential expression analyses were performed in R (55). Lowly expressed genes were filtered using the *filterByExpr* function in the edgeR package (56), with the experimental group specified and all other parameters left at their default values. This method retains genes with sufficient expression based on library sizes and study design, requiring no less than 10 reads in at least the minimum number of samples defined by the smallest group. In our study, this corresponded to retainment of genes with a minimum of 10 reads in at least 24 samples. This filtering strategy reduces noise from low-abundance transcripts and improves statistical power for downstream differential expression analyses (56).

Limma-based RNA-seq analyses require transformation of count data prior to differential expression modeling (57). In the present study, we analyzed RNA-seq data using the limma-voom framework with quality weights, which models the mean-variance relationship of log2-transformed counts at both the observation and sample levels. Read counts were first normalized using the trimmed mean of M-values (TMM) method to account for differences in library size (58, 59). The voom transformation generates precision weights that stabilize variance across expression levels, while quality weights further adjust for between-sample variability. Together, these steps improve the detection of DEGs by accounting for technical and latent sources of variability and yielding approximately normally distributed expression values. The weighted, normalized expression values were used to estimate gene-wise log2 fold change differences for vapers and smokers relative to non-users. Genes identified as differentially expressed in these primary comparisons were further examined in sensitivity analyses to assess the contribution of vaping and smoking dose and vaping product characteristics to the observed expression patterns. In our analyses, we did not filter out long noncoding RNAs (lncRNAs) as there is mounting evidence on the role of lncRNAs as a prime regulator of gene expression in health and disease states (60–63).

### 2.7 Primary model | differential gene expression analysis

DEGs in vapers and smokers relative to non-users were identified using the R/Bioconductor limma package with an empirical Bayes moderated t-test (64), adjusting for age and sex (65). Linear models were fit for each gene i and subject j as:

y_ij_ = β_i_Classification_j_ + δ_i_Age_j_ + µ_i_Sex_j_ + ϵ_ij_.

where Classification_j_ indicates group membership (non-user, vaper, or smoker), Age_j_ represents age in years, and Sex_j_ represents biological sex. Inclusion of age and sex as covariates in the linear model adjusts for their potential confounding effects on gene expression by estimating group effects conditional on these variables. Sex-stratified analyses and interaction effects between sex and group membership were not evaluated. Empirical Bayes smoothing was applied to the standard errors, borrowing information across genes (49–52). Two pre-defined contrasts (vapers vs. non-users, smokers vs. non-users) were evaluated, with the difference in regression coefficients representing log2 fold changes. Genes were considered differentially expressed if they had an absolute fold change greater than 1.5 and a false discovery rate (FDR) below 5%, with multiple testing correction performed using the Benjamini-Hochberg procedure (66). The DEGs served as the basis for downstream sensitivity analyses, examining dose-dependent and product-specific influences on gene expression.

### 2.8 Sensitivity model | ordinal sensitivity analysis

*Post hoc* ordinal sensitivity analyses were conducted to evaluate dose-response relationships between DEGs and vaping or smoking indices. Exposure-specific dose metrics included cumulative e-liquid (mL), cumulative e-nicotine (mg), and years vaped for vaping, and pack-years for smoking. Plasma cotinine concentration was used as an additional exposure dose metric for both vapers and smokers. Cumulative e-liquid was calculated based on self-reported total volume of e-liquid used per one’s lifetime, while cumulative e-nicotine was estimated as the self-reported total nicotine content in e-liquid consumed during one’s lifetime. Pack-year was calculated as the product of the average number of cigarette packs smoked per day and the total number of years smoked (15, 53).

To isolate the association of each dose metric, analyses were conducted separately for vapers and smokers. Within each group, participants were stratified independently for each dose metric into two exposure categories using the group-specific median as the cutoff: low exposure (dose metric below the group-specific median) and high exposure (dose metric at or above the group-specific median). Exposure status was modeled as an ordinal variable with three levels (no exposure [non-user], low exposure, and high exposure), such that the estimated regression coefficient represents the average log2 fold change in gene expression per one-category increase along this ordered exposure scale. Differential expression analysis for these ordinal variables was conducted using the same limma-based framework as in the primary model, adjusting for age and sex.

For vapers, three ordinal sensitivity models were evaluated independently, corresponding to cumulative e-liquid, cumulative e-nicotine, and years vaped. For smokers, an analogous model was applied using pack-years as the exposure metric. Plasma cotinine concentration was evaluated as a further ordinal exposure metric in both vapers and smokers, with categories defined using group-specific median (*i.e.*, vaping-specific for vapers and smoking-specific for smokers), and was modeled separately within each group to assess nicotine-attributable effects within each group. Each model assessed whether DEGs identified in the primary analyses were concordantly expressed with the same directionality (either over- or under-expressed in both analyses) and remained statistically significant when the exposure was stratified into no exposure (non-user), low exposure, and high exposure. Collectively, these analyses evaluated the impact of multiple dose metrics on differential gene expression. DEGs that were concordantly expressed and statistically significant across all dose metrics within a group were designated as *‘common’* DEGs, whereas DEGs that were concordantly expressed and statistically significant for at least one, but not all dose metrics were designated as *‘partial’* DEGs. In this framework, DEGs were evaluated across dose metrics within each group to identify genes with consistent dose-response patterns.

### 2.9 Sensitivity model | nominal sensitivity analysis

*Post hoc* nominal sensitivity analyses were performed to examine the influence of product characteristics on gene expression among vapers. These analyses followed the same limma-based framework as the ordinal sensitivity models, adjusting for age and sex, but focused on categorical factors without an inherent ordering. Specifically, two product characteristics were analyzed, including device generation and e-liquid flavor. Device generation was categorized by the type of vaping device used (first-, second-, or third generation), with an additional category for participants reporting use of multiple device types. E-liquid flavors were classified into five categories, including sweet, fruit, mint/menthol, tobacco, and a combination of different flavors. Although tobacco flavor was defined *a priori* as a category, none of our participants reported use of tobacco-flavored e-liquids; therefore, this category was not represented in downstream analyses.

Similar to the ordinal sensitivity analyses, vapers were stratified by each product characteristic with each category compared to the non-user reference group. Analyses were conducted separately for device generation and e-liquid flavor to determine whether DEGs identified in the primary analyses were concordantly expressed and remained statistically significant across categories of each characteristic. DEGs that were concordantly expressed and statistically significant across all categories of a given product characteristic were designated as *‘common’* DEGs, whereas DEGs that were concordantly expressed and statistically significant in at least one, but not all categories of device generation or of e-liquid flavor were designated as *‘partial’* DEGs. These analyses enabled evaluation of whether vaping product characteristics were associated with differential gene expression beyond dose, highlighting genes whose expression patterns varied across models of device generation and e-liquid flavors.

### 2.10 Gene ontology and functional analyses

Gene lists generated by limma-based RNA-seq (**Supplementary Table 1**, Sheets 1-14) were used for functional enrichment analysis and biological interpretation. Functional identification of canonical pathways, diseases, and upstream regulators was performed using QIAGEN’s Ingenuity Pathway Analysis (IPA v. 2020; QIAGEN Bioinformatics, Redwood City, CA; www.ingenuity.com), as previously described (15, 65). Gene ontology (GO) analysis was performed in Enrichr using gene symbols (67). The top redundant GO terms were slimmed using REVIGO (v1.8.2), with the allowed similarity set to a small threshold of 0.5 and the GO term database set to *Homo Sapiens* (http://revigo.irb.hr).

### 2.11 Statistics

Results are expressed as median (interquartile range [IQR]) for continuous variables and as counts (percentages) for categorical variables. Normality of continuous variables was assessed using the Shapiro-Wilk test and visual inspection of distributions. As data were not assumed to follow a normal distribution, non-parametric methods were used. Continuous variables were compared across groups using the Kruskal-Wallis test. When the overall test was statistically significant, *post hoc* pairwise comparisons (vapers vs. non-users and smokers vs. non-users) were performed using Dunn’s test. Categorical variables were compared using the Chi-squared test or Fisher’s exact test, as appropriate. Spearman rank correlation coefficients were calculated to assess pairwise associations among continuous exposure variables within vapers and smokers. Statistical tests applied for other analyses are specified in the text. All tests were two-sided, and p-values of <0.05 were considered statistically significant. All statistical analyses were performed in R (55). IPA’s built-in statistical procedures were used for all molecular pathways, gene networks, and functional analyses (QIAGEN Bioinformatics, www.ingenuity.com).

## 3 Results

### 3.1 Overview of the study population

Detailed characteristics of the study population are summarized in **Table 1**. The study population included 35 vapers, 24 smokers, and 24 non-users. The median age of vapers, smokers, and non-users was 28.0, 42.0, and 24.5 years, respectively. The sex distribution was similar in vapers and smokers to that of non-users. Among vapers, the median duration of vaping was 3.0 years, with wide variability in cumulative e-liquid and cumulative e-nicotine consumption. Most vapers (60.0%) reported use of third-generation devices, and nearly half (42.9%) reported using multiple e-liquid flavors. Smokers had substantial cumulative tobacco use, with a median of 12.3 pack-years. Plasma cotinine concentrations were statistically significantly higher in both vapers and smokers (median = 113.9 ng/ml and 122.0 ng/ml, resp.) than non-users (median = 2.5 ng/ml) (p<0.001).

**Table 1:** Characteristics of the study population.

Characteristic	Specifics	Vapers (N = 35)	Smokers (N = 24)	Non-users (N = 24)
Age^*^		28.0 (26.0, 36.0) ^‡^	42.0 (30.5, 53.5) ^‡^	24.5 (23.0, 27.0)
Sex^†^				
	Male	28 (80.0%)	19 (79.2%)	15 (62.5%)
	Female	7 (20.0%)	5 (20.8%)	9 (37.5%)
Race^†^		^‡^	^‡^	
	White	14 (40.0%)	5 (20.8%)	3 (12.5%)
	Hispanic	10 (28.6%)	1 (4.2%)	4 (16.7%)
	African American	4 (11.4%)	9 (37.5%)	2 (8.3%)
	Asian	6 (17.1%)	4 (16.7%)	12 (50.0%)
	Other*^§^*	1 (2.9%)	5 (20.8%)	3 (12.5%)
Marital status^†^				
	Single and never married	25 (71.4%)	18 (75.0%)	22 (91.7%)
	Married	2 (5.7%)	3 (12.5%)	0 (0.0%)
	Currently living with someone	2 (5.7%)	0 (0.0%)	0 (0.0%)
	Widowed	1 (2.9%)	0 (0.0%)	0 (0.0%)
	Separated	0 (0.0%)	0 (0.0%)	0 (0.0%)
	Divorced	5 (14.3%)	3 (12.5%)	2 (8.3%)
BMI^*^, *^¶^*		28.5 (23.6, 33.4)	27.2 (23.6, 29.1)	24.4 (21.7, 27.6)
Education^†^		^‡^	^‡^	
	Less than high school	0 (0.0%)	3 (12.5%)	0 (0.0%)
	High school diploma or GED*^#^*	9 (25.7%)	2 (8.3%)	0 (0.0%)
	Some college completed or currently enrolled in college	12 (34.3%)	8 (33.3%)	0 (0.0%)
	College degree or higher	14 (40.0%)	11 (45.8%)	24 (100.0%)
Employment status^†^			^‡^	
	Full time	24 (68.6%)	12 (50.0%)	21 (87.5%)
	Part time	5 (14.3%)	4 (16.7%)	1 (4.2%)
	Retired or disability	1 (2.9%)	3 (12.5%)	0 (0.0%)
	Unemployed	5 (14.3%)	5 (20.8%)	2 (8.3%)
Pretax annual income^†^			^‡^	
	<$30k	16 (45.7%)	14 (58.3%)	14 (58.3%)
	≥$30-<$75k	12 (34.3%)	5 (20.8%)	6 (25.0%)
	≥$75-<$120k	4 (11.4%)	3 (12.5%)	1 (4.2%)
	≥$120k	3 (8.6%)	2 (8.3%)	3 (12.5%)
Years vaped^*^		3.0 (2.0, 5.0)	NA	NA
Years smoked^*^		NA	23.0 (14.0, 32.5)	NA
Pack-year^*^, *^||^*		NA	12.3 (4.7, 22.6)	NA
Plasma cotinine (ng/ml)^*^, ^**^		113.9 (29.4, 129.0) ^‡^	122.0 (50.8, 130.7) ^‡^	2.5 (2.5, 2.5)
Cumulative e-liquid (ml)^*^, ^††^		5,279.0 (3,028.5, 9,100.0)	NA	NA
Cumulative e-nicotine (mg)^*^, ^‡‡^		21,920.3 (6,844.2, 53,235.0)	NA	NA
Device generation^†^, ^§§^				
	1	2 (5.7%)	NA	NA
	2	2 (5.7%)	NA	NA
	3	21 (60.0%)	NA	NA
	Multiple	10 (28.6%)	NA	NA
E-liquid flavor^†^, ^¶¶^				
	Fruit	12 (34.3%)	NA	NA
	Sweet	5 (14.3%)	NA	NA
	Mint or Menthol	3 (8.6%)	NA	NA
	Tobacco	0 (0.0%)	NA	NA
	Multiple	15 (42.9%)	NA	NA
Secondhand smoke exposure^†^, ^##^			^‡^	
	Yes	7 (20.6%)	8 (33.3%)	1 (4.2%)
	No	27 (79.4%)	16 (66.7%)	23 (95.8%)
Air pollution^†^, ^‖^				
	None	7 (20.0%)	5 (20.8%)	5 (20.8%)
	Home or work	13 (37.1%)	11 (45.8%)	9 (37.5%)
	Home and work	15 (42.9%)	8 (33.3%)	10 (41.7%)
Grilled/broiled/roasted food intake (times/week)^†^, ^***^				
	<3 times/week	19 (54.3%)	14 (58.3%)	17 (70.8%)
	≥3 times/week	16 (45.7%)	10 (41.7%)	7 (29.2%)

^*^ Results are expressed as Median (Q1, Q3).

^†^ Numbers and percentages (inside brackets) are indicated.

^‡^ Statistically significant as compared to non-users, p < 0.05.

^§^ Other = Multiracial or Native American

^¶^ BMI = Body mass index [Weight (kg) ÷ Height (m)²]

^#^ GED = General Education Development or General Education Diploma, The GED or High School Equivalency Certificate shows that one has a level of knowledge equivalent to a high school graduate.

^‖^ Pack-year is calculated by multiplying the number of packs of cigarettes a person smoked per day by the number of years he or she smoked.

^**^ Plasma cotinine concentrations were measured using a solid-phase competitive ELISA (Abnova Corp.).

^††^ Cumulative e-liquid is calculated as the total volume of e-liquid (in milliliters) vaped by a person during his or her lifetime.

^‡‡^ Cumulative e-nicotine was calculated as the total amount of nicotine (in milligrams) present in e-liquid vaped by a person during his or her lifetime.

^§§^ Device types are divided into 1st Generation: Cig-a-Like, disposable; 2nd Generation: Vape Pen, mid-size (laser pointer) with pre-filled or re-fillable cartridges; 3rd Generation: Mod or Tank, large size; and Multiple (a combination of different generation devices).

^¶¶^ E-liquid flavor are divided into five categories: Sweet; Fruit; Mint/Menthol; Tobacco; and multiple (a combination of a different flavors).

^##^ Secondhand smoke exposure was assessed using questionnaires eliciting information on household and workplace smoking status.

^‖^ Air pollution was assessed using questionnaires querying air quality at place of residence and work, e.g., living or working within four blocks of highways or proximity to pollution sources, such as industrial sites.

^***^ Consumption of grilled/broiled/roasted food was evaluated using a food frequency questionnaire and results are expressed as “<3 or ≥3 times/week”.

NA = Not applicable.

### 3.2 Primary model | genome-wide differential gene expression analysis

**Figure 1:**
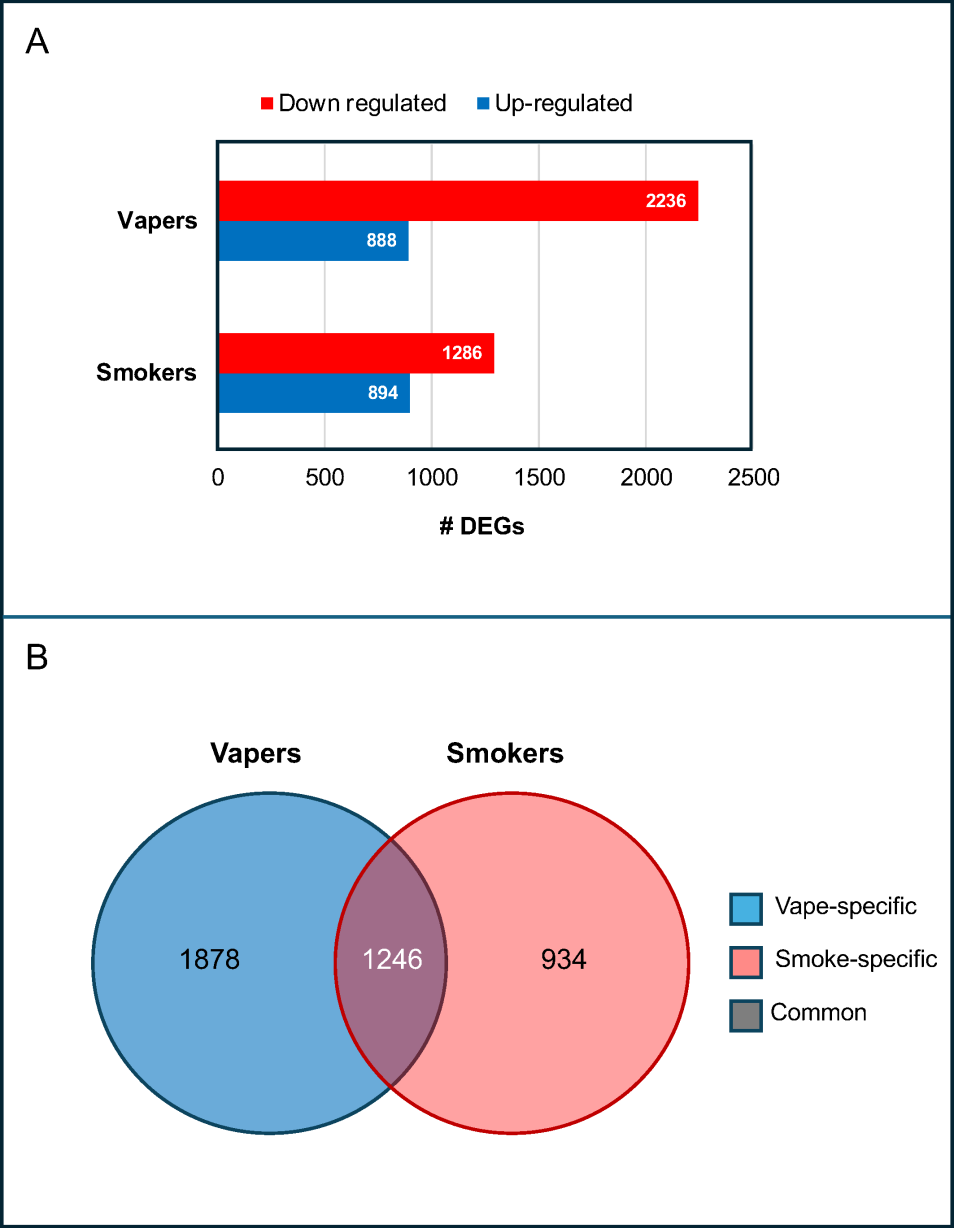
Differential expression of genes detected by RNA-seq in vapers and smokers as compared to non-users. **(A)** Numbers of up-regulated and down-regulated genes in vapers and smokers are indicated (FC > 1.5 and FDR < 0.05). **(B)** Venn diagram of differentially expressed genes (DEGs) in vapers and smokers.

To evaluate the impact of vaping and smoking on global gene expression, we performed limma-based RNA-seq analysis (51) on total RNA isolated from oral epithelial cells. Both vapers and smokers exhibited substantial transcriptomic responses. There were 3,124 DEGs identified in vapers as compared to non-users (fold change > 1.5, FDR < 0.05) (**Figure 1A**). Of these, 888 genes (28.4%) were up-regulated and 2,236 (71.6%) were down-regulated. In smokers, 2,180 DEGs were identified as compared to non-users, with 894 genes (41.0%) being up-regulated and 1,286 (59.0%) being down-regulated. Complete DEG lists are provided in **Supplementary Table 1** (Sheet 1: vapers; Sheet 2: smokers).

The DEGs identified in vapers and smokers can be classified into three categories: (I) vape-specific, defined as genes differentially expressed exclusively in vapers; (II) smoke-specific, defined as genes differentially expressed exclusively in smokers; and (III) common, defined as genes differentially expressed in both vapers and smokers. Vape-specific DEGs accounted for 60.1% of all DEGs in vapers (1,878/3,124), whereas smoke-specific DEGs comprised 42.8% all DEGs in smokers (934/2,180) (**Figure 1B**). Common DEGs represented 39.9% and 57.2% of all DEGs in vapers and smokers, respectively.

### 3.3 Ordinal sensitivity analysis | dose-dependent differential gene expression

To assess the effect of vaping and smoking dose on gene expression levels, we compared DEGs identified in the primary analysis (primary DEGs) with those detected in the ordinal sensitivity models using exposure-specific metrics (**Table 2**). In vapers, 980 (31.4%), 1,378 (44.1%), and 1,329 (42.5%) of the 3,124 primary DEGs showed statistically significant, concordant expression (*i.e.*, with same directionality) when examined in the cumulative e-liquid, cumulative e-nicotine, and years vaped sensitivity models, respectively (**Table 2**; **Supplementary Table 1**, Sheets 3-5). Using plasma cotinine as another dose metric, 50.5% of primary DEGs in vapers showed statistically significant, concordant expression in the sensitivity analysis (**Supplementary Table 1**, Sheet 6). Notably, common DEGs (statistically significant across all dose metrics) comprised 27.6% of primary DEGs in vapers, whereas partial DEGs (statistically significant for at least one, but not all dose metrics) comprised 28.8% (**Table 2**; **Supplementary Figure 1A**).

**Table 2:** Concordance of primary differentially expressed genes (DEGs) across ordinal and nominal sensitivity models.

Group	Analysis	Exposure index	Specifics	Number of differentially expressed genes (percentage)
Vapers				
	Primary model			3124 (100%)
	Ordinal sensitivity model			
		Cumulative e-liquid		980 (31.4%)
		Cumulative e-nicotine		1378 (44.1%)
		Years vaped		1329 (42.5%)
		Plasma cotinine		1578 (50.5%)
		Common^*^		862 (27.6%)
		Partial^†^		900 (28.8%)
	Nominal sensitivity model			
		Device generation		
			1	0 (0%)
			2	0 (0%)
			3	1747 (55.9%)
			Multiple	1367 (43.8%)
			Common^*^	0 (0%)
			Partial^†^	1907 (61%)
		E-liquid flavor		
			Sweet	92 (2.9%)
			Fruit	970 (31%)
			Mint or Menthol	27 (0.9%)
			Multiple	2009 (64.3%)
			Common^*^	0 (0%)
			Partial^†^	2080 (66.6%)
Smokers				
	Primary model			2180 (100%)
	Ordinal sensitivity model			
		Pack-year		1241 (56.9%)
		Plasma cotinine		1307 (60%)
		Common^*^		1180 (54.1%)
		Partial^†^		188 (8.6%)

^*^ Differentially expressed genes that showed statistically significant concordant expression across all dose metrics or levels of a product characteristic within a group.

^†^ Differentially expressed genes that showed statistically significant concordant expression for at least one, but not all dose metrics or levels of a product characteristic within a group.

In smokers, 1,241 of 2,180 primary DEGs (56.9%) showed statistically significant, concordant expression in the pack-year sensitivity analysis (**Table 2**; **Supplementary Table 1**, Sheet 7), whereas 1,307 of 2,180 (60.0%) showed statistically significant, concordant expression in the plasma cotinine sensitivity analysis (**Table 2**; **Supplementary Table 1**, Sheet 8). Overall, a larger proportion of common DEGs was observed in smokers compared to vapers (54.1% vs. 27.6%, respectively). Partial DEGs comprised 8.6% of the primary DEGs in smokers, which is lower than that in vapers (28.8%) (**Table 2**; **Supplementary Figure 1B**).

To characterize relationships among dose metrics, we performed correlation analyses, which showed a strong positive association between cumulative e-liquid and cumulative e-nicotine consumptions (Spearman ρ=0.75, p<0.001) (**Supplementary Figure 2A**). In contrast, plasma cotinine levels were weakly correlated to cumulative exposure dose metrics in both vapers and smokers (**Supplementary Figures 2A, B**). The latter is consistent with the fact that cotinine is a validated marker of recent rather than long-term exposure to nicotine-containing tobacco products (both for combustible cigarettes and e-cigs) (9, 54).

**Figure 2:**
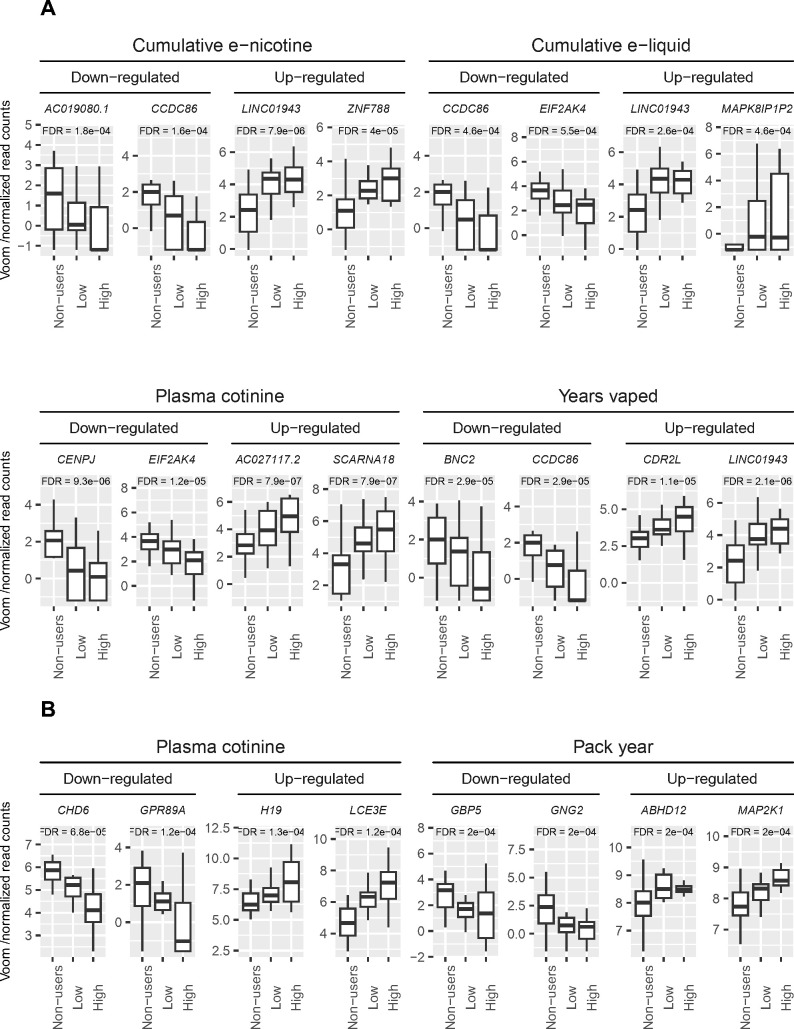
Visualization of the results of ordinal sensitivity analyses. Gene expression results for the four most significantly differentially expressed (two down-regulated and two up-regulated) DEGs for vapers **(A)** and smokers **(B)**, as determined by ordinal sensitivity analyses, are shown. In the box and whisker plots, the ‘lower’ and ‘upper’ edges of boxes represent the 1st and 3rd quartiles, respectively (25 and 75 percentiles, respectively). Horizontal lines within the boxes represent the medians (2nd quartile or 50 percentile). The ‘lower’ and ‘upper’ vertical lines extending from the boxes, also known as “whiskers”, represent the lowest and highest data points, respectively, excluding outlier(s).

To illustrate the dose-dependent transcriptional changes, we visualized DEGs identified in the ordinal sensitivity analyses. **Figure 2** displays a representative subset of these genes, specifically the top four DEGs (two up-regulated and two down-regulated) within each group for each exposure metric. Among vapers, these genes exhibited clear monotonic increases or decreases in expression across ordinal exposure categories for cumulative e-liquid, cumulative e-nicotine, years vaped, and plasma cotinine, although the magnitude of these trends varied by metric. The top DEGs among smokers demonstrated consistently monotonic dose-response patterns across both pack-year and plasma cotinine metrics.

### 3.4 Nominal sensitivity analysis | product-specific differential gene expression

To explore the influence of vaping product characteristics on gene expression, we compared primary DEGs with those detected in the nominal sensitivity models (**Table 2**). Among vapers, of the 3,124 primary DEGs, statistically significant, concordant differential expression was detected only in the third- and multiple-generation device models (**Table 2**; **Supplementary Table 1**, Sheets 9-10). No DEGs were identified in the first- or second-generation device models, likely reflecting limited sample sizes within these categories; consequently, there were no common DEGs across all models of device generation. However, when only third- and multiple-generation device models were considered, common DEGs (statistically significant across both models) comprised 58.0% of primary DEGs in vapers (**Supplementary Figure 1C**). Partial DEGs (statistically significant for at least one, but not all models of device generation) comprised 61.0% of DEGs detected in vapers in the primary analysis (**Table 2**).

With respect to e-liquid flavor, 92 (2.9%), 970 (31.0%), 27 (0.9%), and 2,009 (64.3%) of the 3,124 primary DEGs in vapers were concordantly expressed and statistically significant in the sweet, fruit, mint/menthol, and multiple flavor sensitivity models, respectively (**Table 2**; **Supplementary Table 1**, Sheets 11-14). Notably, there were no common DEGs across all e-liquid flavors. However, partial DEGs (statistically significant in at least one, but not all e-liquid flavors) comprised 66.6% of primary DEGs in vapers (**Table 2**; **Supplementary Figure 1D**).

**Figure 3:**
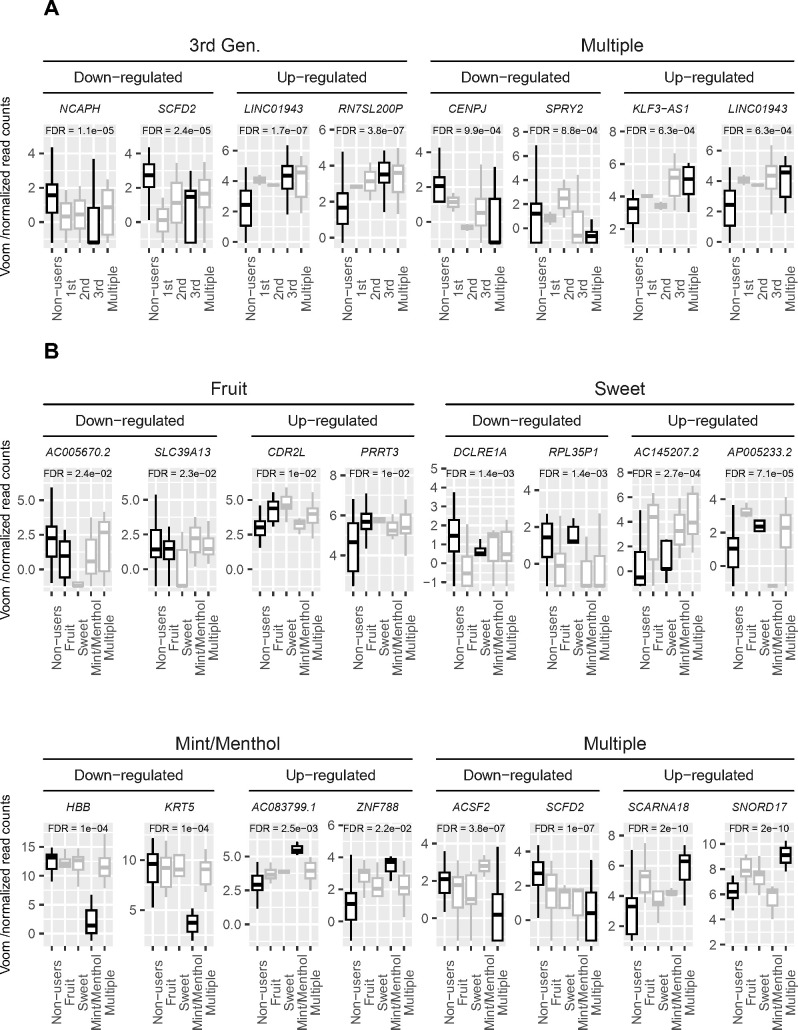
Visualization of the results of nominal sensitivity analyses. Gene expression results for the four most significantly differentially expressed (two down-regulated and two up-regulated) DEGs for vapers, as determined by nominal sensitivity analyses, are shown. In the box and whisker plots, the ‘lower’ and ‘upper’ edges of boxes represent the 1st and 3rd quartiles, respectively (25 and 75 percentiles, respectively). Horizontal lines within the boxes represent the medians (2nd quartile or 50 percentile). The ‘lower’ and ‘upper’ vertical lines extending from the boxes, also known as “whiskers”, represent the lowest and highest data points, respectively, excluding outlier(s).

To characterize transcriptional response across product characteristics, we visualized DEGs identified in the nominal sensitivity analyses. **Figure 3** displays a representative subset of these genes, specifically the top four DEGs (two up-regulated and two down-regulated) within each category of device generation and e-liquid flavor. Among vapers, these genes exhibited distinct expression patterns across product categories, with consistent directionality of change relative to non-users. However, the magnitude of these changes differed substantially by product characteristic and by category, consistent with the limited number of common DEGs observed. Notably, DEGs associated with higher-generation devices and use of multiple e-liquid flavors showed the most pronounced and consistent expression changes (see, **Supplementary Table 1** [Sheets 9, 11-13] and **Figure 3**), whereas earlier-generation and single-flavor categories showed fewer or more variable transcriptional responses (see, **Supplementary Table 1** [Sheets 10, 14] and **Figure 3**).

### 3.5 Functional enrichment analyses

**Figure 4:**
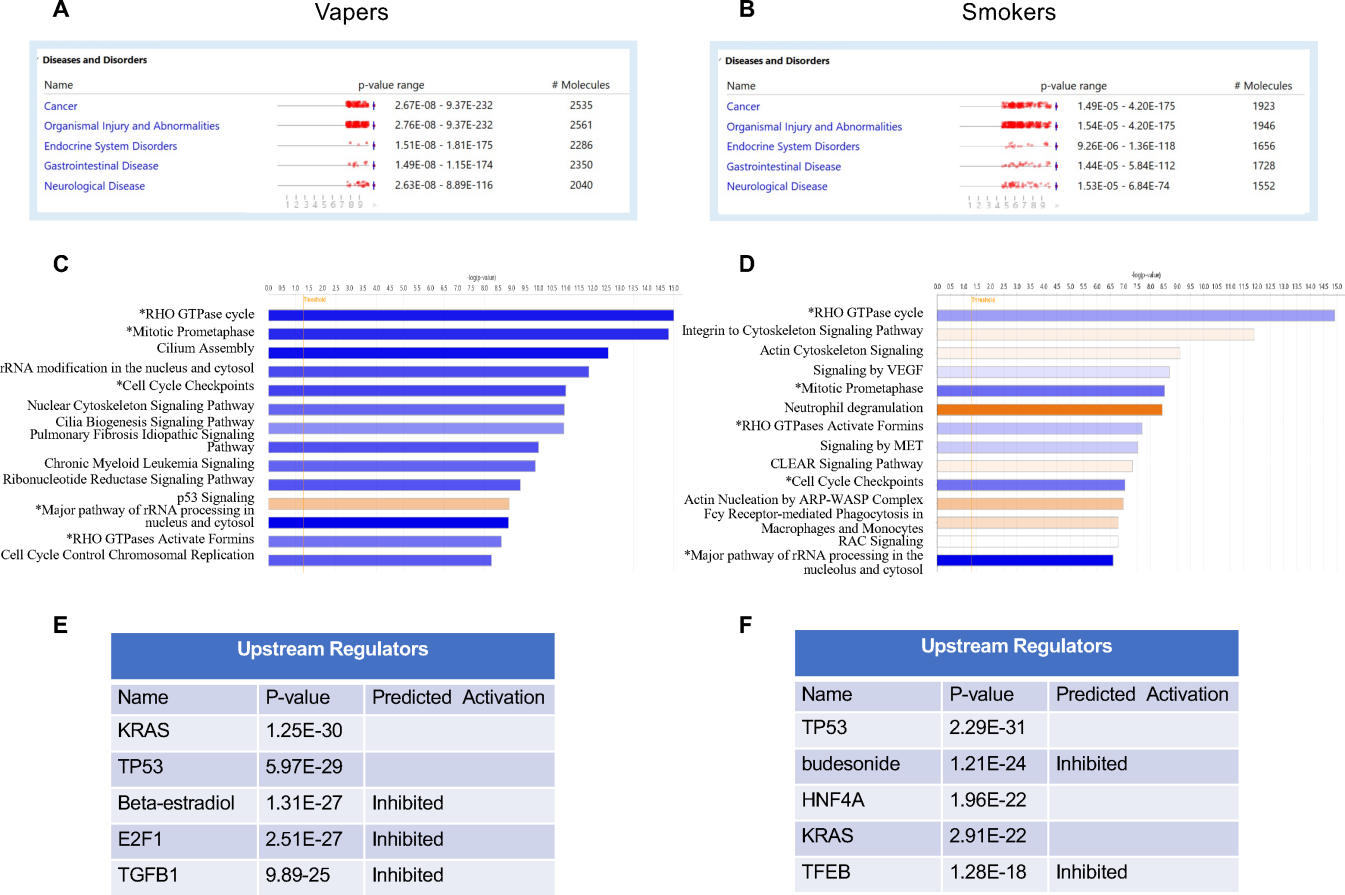
Functional pathway and upstream regulator analysis of DEGs identified by the primary model in vapers and smokers as compared to non-users. **(A, B)** Diseases and functions identified by IPA in vapers and smokers. In both groups, DEGs were predominantly associated with “cancer”. **(C, D)** Top canonical pathways identified by IPA in the two datasets. (*) Common pathways. The orange and blue colored bars indicate predicted pathway activation, or predicted inhibition, respectively (z-score). White bars, z-score close to 0. Gray bars, no prediction. **(E, F)** Upstream Regulator Analysis of DEGs in vapers and smokers. IPA’s Upstream Regulator Analysis was used to identify upstream regulators that may be responsible for the gene expression changes observed in the two groups.

We used IPA to obtain detailed gene enrichment information on the DEG lists generated by the primary model in vapers and smokers as compared to non-users (**Figure 1**; **Table 2**; **Supplementary Table 1**, Sheets 1-2). Of the 3,124 aberrantly expressed genes in vapers, 2,791 (89.3%) had an assigned ID and passed the cutoffs for subsequent analysis. In smokers, 2,073 out of 2,180 DEGs (95.1%) mapped to known IDs and were further processed in IPA. As shown in **Figure 4**, cancer was the top disease associated with the aberrantly expressed genes in both vapers (2,535/2,791: 90.8%) and smokers (1,923/2,073: 92.8%) (**Figures 4A, B**). As previously described by us and others (15, 68, 69), the ‘RHO GTPase Cycle’ was the most affected pathway identified in both vapers and smokers (**Figures 4C, D**). Other affected signaling pathways, common to both vapers and smokers, included the ‘Mitotic Prometaphase’, which regulates nuclear envelope breakdown and microtubule attachment (70, 71), the ‘rRNA modification in the nucleolus and cytosol’, which is responsible for processing and maturation of ribosomal RNA (72), and the ‘Cell Cycle Checkpoints’, which monitors error-free DNA replication and division (71). Several canonical pathways were also unique to either the vaper or smoker group. For example, vaping affected ciliogenesis and associated regulatory pathways as well as chromosomal replication, while smoking altered the ‘Vascular Endothelial Growth Factor’ (VEGF) signaling and the neutrophil degranulation pathway (**Figures 4C, D**). We also used IPA to identify the upstream regulators (*i.e.*, transcription factors, kinases, chemicals, drugs, etc.) that may affect downstream targets and drive the gene expression changes observed in the two datasets. Amongst the top upstream regulators, *p53* and *KRAS* (73, 74) were identified based on statistical significance, although there was no clear prediction of them being either activated or inhibited (**Figures 4E, F**).

**Figure 5:**
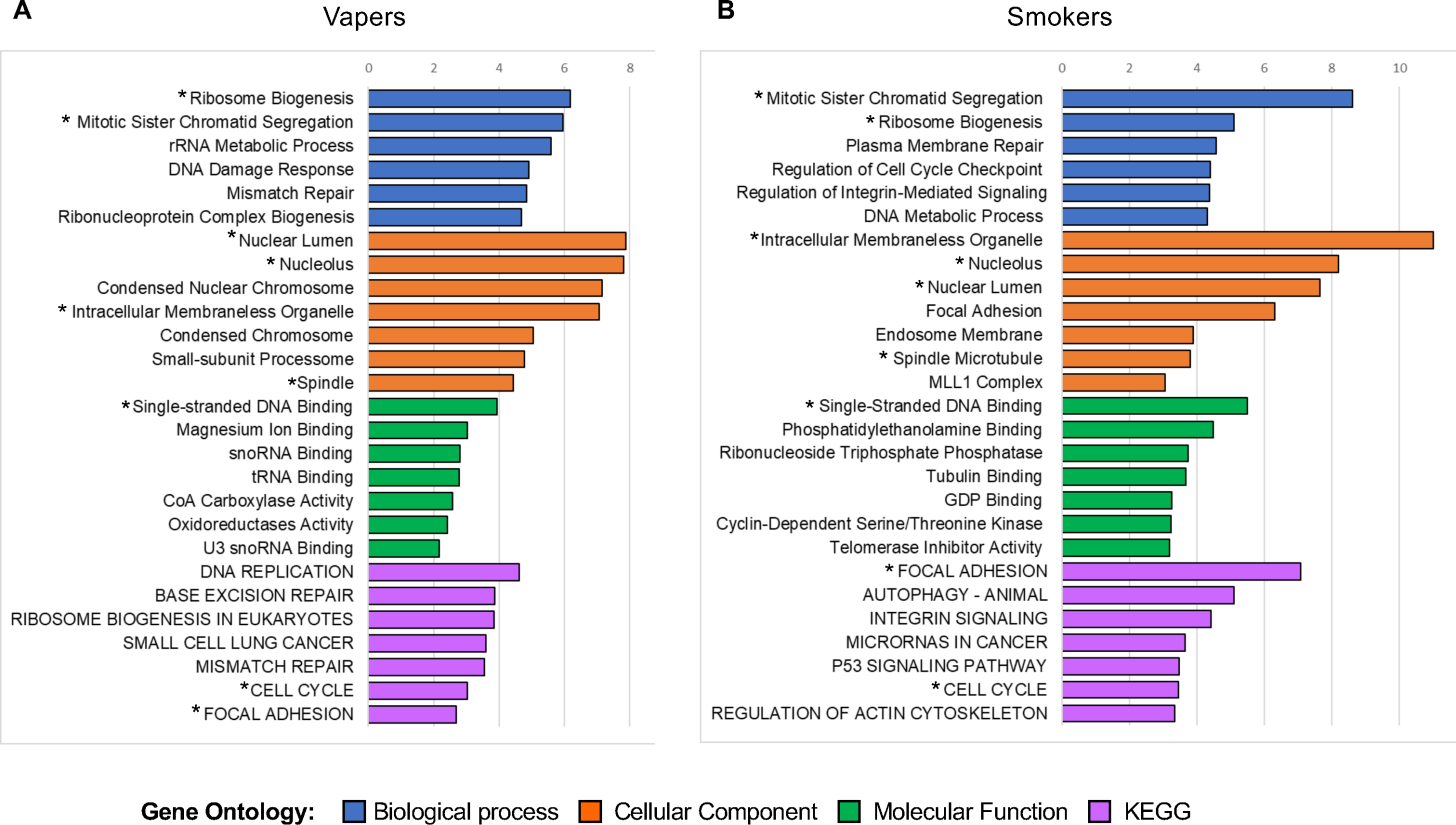
GO and KEGG analysis of DEGs identified by the primary model in vapers **(A)** and smokers **(B)** as compared to non-users. Enrichment analysis was performed using Enrichr and top redundant GO terms were slimmed using REVIGO. (*) Common terms.

We also used GO analysis to highlight the biological processes, cellular components and molecular functions that were enriched in vapers and smokers (**Figures 1A, B**). The top significant slimmed GO terms identified by Enrichr are listed in **Figure 5**. In accordance with IPA results, vapers and smokers shared many GO terms, including ‘ribosome biogenesis’ and ‘mitotic sister chromatid segregation’ in the biological process category; ‘nuclear lumen’, ‘nucleolus’, ‘intracellular membraneless organelle’ and ‘spindle’ in the cellular component category; and ‘single-stranded DNA binding’ in the molecular function category. ‘Cell cycle’ and ‘focal adhesion’ were also common biological pathways identified by Kyoto Encyclopedia of Genes and Genomes (KEGG) analysis (**Figures 5A, B**). GO and KEGG analyses also detected processes, functions and pathways that were unique to either vapers or smokers. In vapers, the most enriched terms and pathways were associated with ‘DNA damage response’ (both mismatch and base excision repair), ‘snoRNA binding’ and ‘DNA replication’ (**Figure 5A**). In smokers, the most represented terms included ‘regulation of integrin-mediated signaling’, ‘endocytosis’ and ‘autophagy’ (**Figure 5B**).

Furthermore, we used Comparison Analysis in IPA to identify trends or similarities and differences across datasets (primary DEGs) in vapers and smokers. We identified the Aryl Hydrocarbon Receptor (AHR) signaling pathway as one of the canonical pathways impacted in both groups (**Supplementary Figure 3** and **Supplementary Figure 4**). AHR is a ligand-activated transcription factor known to be activated by smoke toxicants (*e.g.*, polycyclic aromatic hydrocarbons) in the cytosol of oral epithelial cells (16). AHR can then translocate to the nucleus where it induces detoxification enzymes (*e.g.*, CYP1A1 and CYP1B1), thus providing a first line of defense against environmental carcinogens (16, 75). Our comparative analysis of the AHR canonical pathway showed dysregulation of 31 genes in vapers versus 17 genes in smokers. Common affected molecules included *HSP90AA1*, *CDKN1B*, *CCND1*, *ALDH18A1*, *ALDH7A1*, *MYC*, *ATM*, *ATR*, *CDK4*, *POLA1*, and *CCNA2* (**Supplementary Figure 4**). We note that although the total number of DEGs belonging to the AHR pathway was higher in vapers than smokers (31 vs. 17), the number of affected molecules in this pathway (based on prediction analysis of activation or inhibition in IPA) was much higher in smokers relative to vapers (**Supplementary Figure 3**). Visual inspection of **Supplementary Figure 3** shows that there are many more color-coded molecules (predicted to be activated [orange nodes] or inhibited [blue nodes]) in the AHR pathway in smokers (panel ‘B’, right side) than vapers (panel ‘A’, left side).

Altogether, these findings are consistent with our recent ‘omics’ studies (15, 30, 65), in which we demonstrated that vapers and smokers show partly similar and partly distinct patterns of gene dysregulation, involving both shared and unique molecular pathways and functions.

## 4 Discussion

We performed transcriptome analysis in oral epithelial cells from vapers and cigarette smokers as compared to non-users, with a specific focus on the impact of vaping and smoking dose as well as vaping product characteristics on gene regulation. Given that oral epithelial cells are a major target for tumor development and other diseases and anomalies associated with tobacco use, and over 90% of all human cancers are of epithelial origin (15, 76–78), the oral epithelium represents a biologically relevant target tissue for investigating early molecular perturbations associated with nicotine-related exposures (30). Using a limma-voom-based analysis of RNA-seq data with adjustment for age and sex and control of the FDR, we identified significant dysregulation of genes in both vapers and smokers relative to non-users. Although comparison of DEGs identified in vapers and smokers showed notable overlap, a large portion of DEGs identified in vapers (60.1%) was not shared with smokers. The DEGs shared between the two groups suggest that some vaping-associated transcriptional changes may reflect mechanisms also affected by combustible cigarette smoke exposure, whereas vape-specific DEGs highlight biological pathways potentially unique to e-cig use. These findings underscore that while vaping and smoking share certain transcriptomic signatures, there are additional, distinct perturbations that warrant independent evaluation.

The above findings were confirmed by our GO and functional analyses whereby we identified the canonical pathways, biological processes and diseases and functions that were altered in vapers and smokers. Consistent with prior studies (15, 30, 68), we observed that cancer was the top disease associated with the dysregulated genes in both vapers (90.8%) and smokers (92.8%) (**Figures 4A, B**). In confirmation, we identified the RHO GTPase pathway as the most disrupted pathway in both vapers and smokers (**Figures 4C, D**). The RHO family of GTPases is a family of small proteins involved in the regulation of many cellular processes relying on the dynamic reorganization of the cytoskeleton, *i.e.*, cell migration, cell adhesion, cell division, establishment of cellular polarity and intracellular transport, all of which are critical for cancer invasion and metastasis. Altered expression of RHO GTPases has been reported in a variety of human malignancies (including tobacco-related cancers) as well as in infectious, immunological, and neurodegenerative diseases (79–81). Other relevant pathways affected in both vapers and smokers include regulation of cell cycle progression, DNA replication and chromosome segregation, all of which show overall inhibition, thus emphasizing the cells’ attempt to reduce mitotic activity and maintain genomic stability following exposure (19, 82) (**Figures 4C, D**). Examination of the canonical pathways that were modulated in either vapers or smokers identified the ‘cilia biogenesis and assembly’ and the ‘integrin signaling’ as the most impacted pathways in vapers and smokers, respectively (**Figures 4C, D**; **Figure 5B**). Ciliogenesis is closely coordinated with the cell cycle and defects of this signaling pathway may represent an early signature event during oncogenic transformation (83). Exposure to unflavored e-cigarettes has been shown to disrupt ciliary function in airway cells by altering the phosphorylation of proteins involved in RHO GTPase signaling, thus leading to impaired mucociliary clearance (84). In line with our previous studies (15, 30), we also confirmed that the integrin signaling pathway, known to modulate cell adhesion, survival, migration and differentiation, was one of the top dysregulated pathways in smokers. Defects in this pathway can promote tumor invasion and metastasis (85–87).

To delineate the drivers of transcriptional changes associated with vaping and smoking, we examined the extent to which various dose metrics explained differential gene expression patterns in vapers and smokers as compared to non-users. Among vapers, 27.6% of DEGs were classified as ‘common’ across all dose metrics (*i.e.*, cumulative e-liquid, cumulative e-nicotine, years vaped, and plasma cotinine). Smokers exhibited a much higher proportion of DEGs (54.1%) classified as common, indicating that smoking-associated transcriptional responses are largely dose-driven and consistent across dose metrics. This pattern aligns with well-established dose-response relationships between smoking intensity or duration and molecular damage or disease risk in epithelial tissues, whereby greater dose is associated with increased biological perturbations (88–91). Although the definition of common DEGs, requiring statistical significance across all dose metrics within a group, imposes a more stringent criterion in vapers (4 metrics in vapers vs. 2 metrics in smokers), the magnitude of this difference suggests that smoking dose metrics capture a more consistent underlying exposure signal, whereas vaping dose metrics may reflect more heterogeneous exposure patterns. The observed higher proportion of ‘partial’ DEGs in vapers than smokers (28.8% vs. 8.6%, respectively; **Table 2**) further corroborates that vaping-related transcriptional responses are less uniformly captured across exposure dose metrics. Together, these findings indicate that dose provides a less unified descriptor of vaping-associated gene expression changes than of smoking-associated transcriptional dysregulation (see, also discussion below).

In addition to dose metrics, we assessed the contribution of product characteristics to vaping-associated transcriptional patterns. Analyses stratified by device generation and e-liquid flavor revealed no common DEGs across categories of either product characteristic, whereas a substantial proportion of DEGs were classified as partial in both product-specific sensitivity analyses. The absence of common DEGs indicates that no single, shared transcriptional signature reflects product-specific characteristics, defined by device generation or e-liquid flavor. Instead, the predominance of partial DEGs suggests that different product characteristics capture partially distinct components of gene expression. When restricting analyses to the more prevalent device models (third- and multiple-generation devices), a high proportion of DEGs (58.0%) were common, suggesting more uniform transcriptional responses among these device types. Nonetheless, we should note that limited statistical power within strata may have reduced the ability to detect common DEGs. In addition to the variable dose-dependent exposure paradigm, the findings further support that the variability extends to vaping product characteristics and their effect on transcriptional regulation. Taken together, the varying dose-dependent and product-specific exposure paradigm implies that neither dose nor product characteristics alone fully account for observed transcriptional responses in vapers. The predominance of partial DEGs across both dose and product characteristics suggests that vaping-associated gene expression changes reflect overlapping but distinct dimensions of exposure, likely influenced by variability in usage rates, device types and features, e-liquid composition, and individual vaping behaviors.

While we underscore the strengths of our study, we also acknowledge several limitations. A major strength is the use of a well-characterized study population with detailed demographic and lifestyle information obtained through standardized questionnaires and interviews. We accounted for relevant biological variables, including age and sex, in all analyses, strengthening internal validity. In addition, we verified that distribution of potential confounding factors, such as air pollution and secondhand smoke exposure, was not statistically significantly different between vapers and non-users. Furthermore, we applied a rigorous, best-practice RNA-seq analytical framework using covariate-adjusted limma-voom modeling with FDR control (50). The genome-wide design, coupled with parallel evaluation of vaping and smoking dose metrics and vaping product characteristics, enabled a multidimensional and unbiased assessment of exposure-associated transcriptional perturbations. Several limitations merit consideration. First, the greater number of primary DEGs identified in vapers compared with smokers differs from some prior reports. However, earlier studies applied less stringent discovery criteria, including unadjusted p-value thresholds or fold change cutoffs without consistent covariate adjustment or multiple testing correction (15, 52, 64, 92). Differences in sequencing depth, pre-processing pipelines, cohort characteristics, and tissue sources further limit direct comparisons (93–95). In addition, although age and sex were included as covariates in our models, we did not evaluate age- or sex-specific effects or interactions, which may also contribute to differences in findings across studies. We note that the latter was beyond the scope and limitations of the present study as it would require a much larger sample size to allow sufficient statistical power to produce meaningful results. Together, variation in reported DEG counts across studies likely reflects methodological and population differences rather than fundamental discrepancies in exposure-associated biology. Second, as compared to vaping dose metrics, smoking dose metrics demonstrated relatively uniform effects on gene expression; however, interpretation of these findings should consider the difference in exposure duration between groups. In our cohort, smokers had a markedly longer median duration of use (23.0 years) as compared to vapers (3.0 years), which may have contributed to the more consistent dose-response patterns observed in smokers. Given that longer exposure duration is associated with cumulative biological effects (26, 96–98), this difference may somewhat limit direct comparisons of dose-related transcriptional changes between vapers and smokers. It is important, however, to note that e-cigs are a relatively new tobacco product as compared to combustible cigarettes (1, 2). Whilst our cohort represented the distribution of vapers and smokers in our catchment area at the time of enrollment into the study, future studies are more likely to recruit vapers and smokers with relatively comparable use duration. If current trends of tobacco product use continue, long-term e-cig users will likely represent a substantial portion of the vaping population. Thus, it will be more feasible for future research studies to recruit participants who have used e-cigs for a longer duration of time, which may near that of average smokers. Future studies should also examine whether product-specific characteristics, such as brand, tar content, mentholation, and filter design further modulate transcriptomic changes in smokers. Third, among vapers, certain exposure subgroups, particularly users of earlier-generation devices, had modest sample sizes, which may have limited statistical power to detect differential expression of genes with smaller effect sizes. Additionally, fourth-generation e-cig device users were not represented in our cohort. At the time of subject recruitment for this study, these devices had only recently emerged and were not yet widely adopted. Hence, the characteristics of e-cig users in our cohort reflect prevalent vaping trends and devices when the study was conducted. Given the continued evolution of vaping technology, future studies in larger population of vapers who use contemporary devices, will be warranted to evaluate whether newer device types produce similar or distinct transcriptional signatures.

In conclusion, we demonstrate that both vaping and smoking are associated with substantial transcriptomic perturbations in oral epithelial cells, but the structure of these perturbations differs fundamentally between exposure types. Smoking-associated gene expression changes appear largely unified across dose metrics, whereas vaping-associated transcriptional responses are distributed across both dose and product-specific dimensions. Our findings suggest that vaping exposure cannot be fully characterized by dose alone and that device configuration and e-liquid formulation represent important contributors to the biological effects of e-cig use. Incorporating multidimensional exposure metrics into future molecular and epidemiologic studies may improve risk stratification and mechanistic understanding. Our data have potential relevance for clinical practice as they provide molecular evidence that may help clinicians better understand and counsel patients about the biological impacts of vaping as compared to smoking. At the population level, identification of exposure-associated transcriptional signatures may also inform public health strategies and contribute to the scientific evidence base considered by regulatory agencies, including the U.S. Food and Drug Administration (FDA), when evaluating the risks of tobacco and nicotine products. In particular, identification of product characteristics and usage patterns associated with transcriptional dysregulation provides data that can inform regulatory decision-making. Regulatory frameworks that account not only for patterns of use but also for device design features and e-liquid formulation may be better positioned to address the biological complexity of e-cig exposure and to support the FDA’s mandate to protect public health.

## Data Availability

The RNA-seq data presented in the study are deposited in the the Gene Expression Omnibus (GEO) repository, and accession number is GSE330022.
