# Supplementary figures and images for: Multidimensional exposure architecture shapes vaping-associated transcriptomic dysregulation in oral epithelium
Source: Front Oncol. 2026 Jun 1;16:1838256. doi: 10.3389/fonc.2026.1838256 (PMC13265642; doi:10.3389/fonc.2026.1838256)

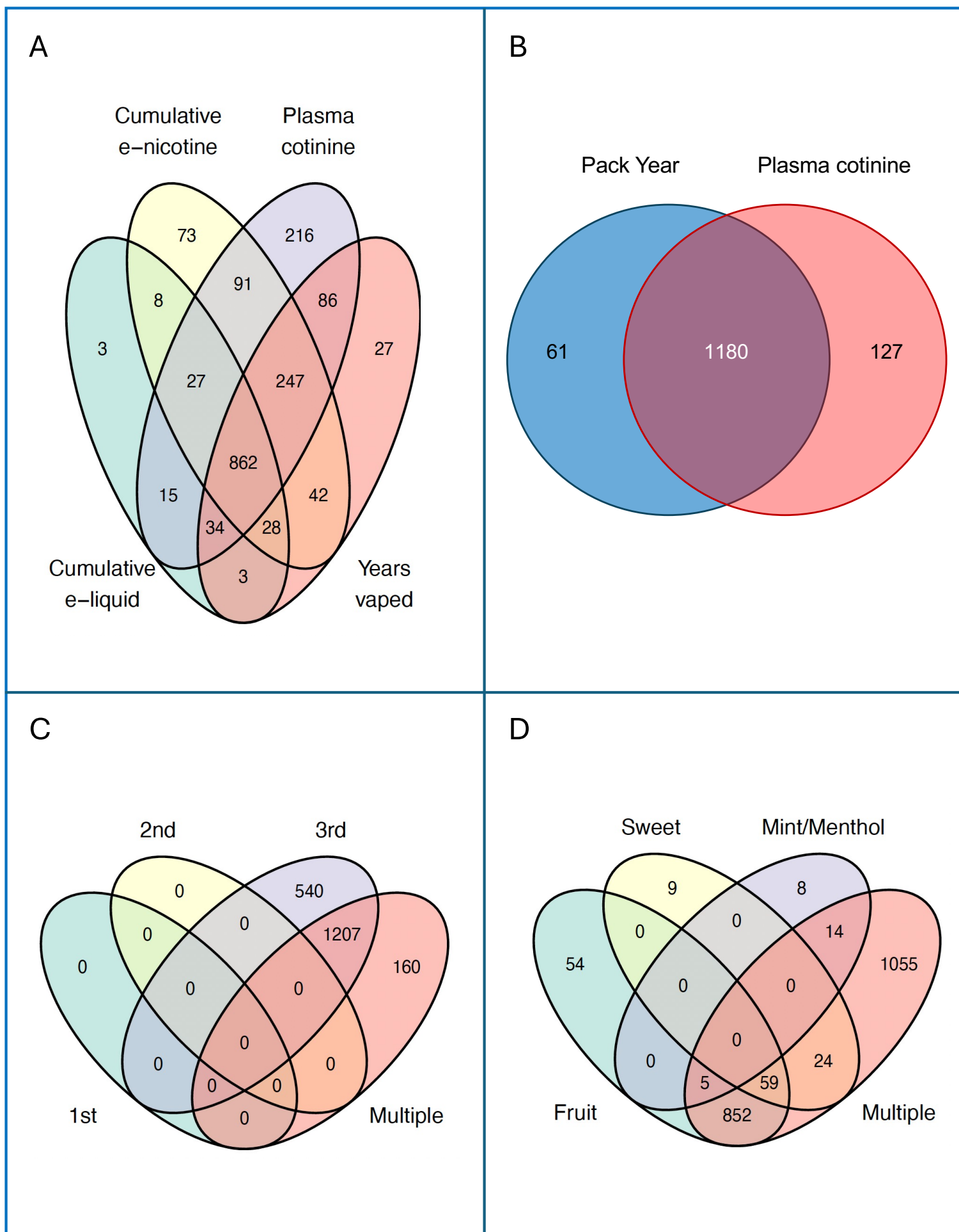

Fig. S1

Supplement: Supplementary file 1 [file DataSheet1.pdf]

**A**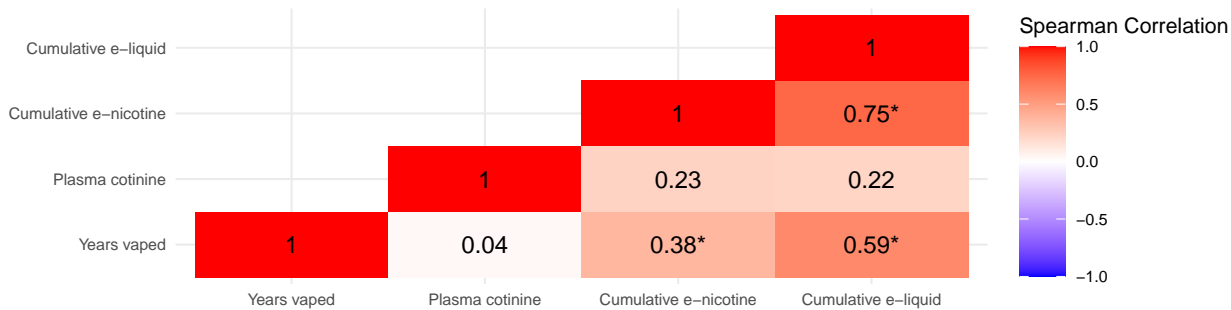

\* = Statistically significant,  $p < 0.05$ .

**B**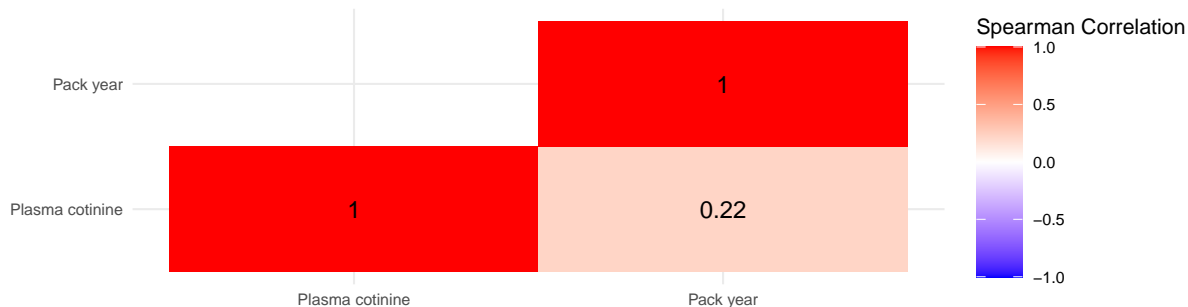

\* = Statistically significant,  $p < 0.05$ .

Fig. S2

Supplement: Supplementary file 2 [file DataSheet2.pdf]

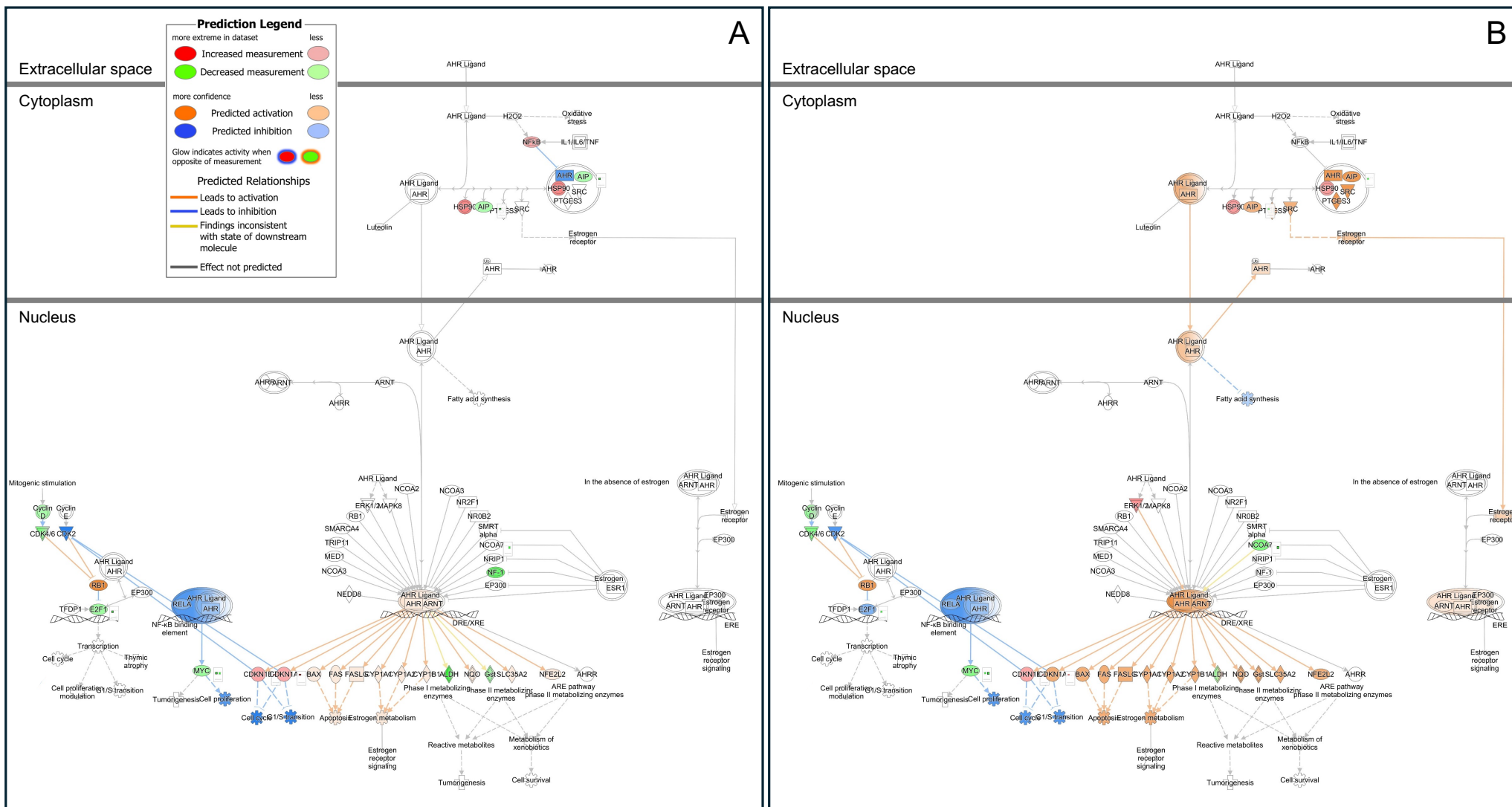

Fig. S3

Supplement: Supplementary file 3 [file DataSheet3.pdf]

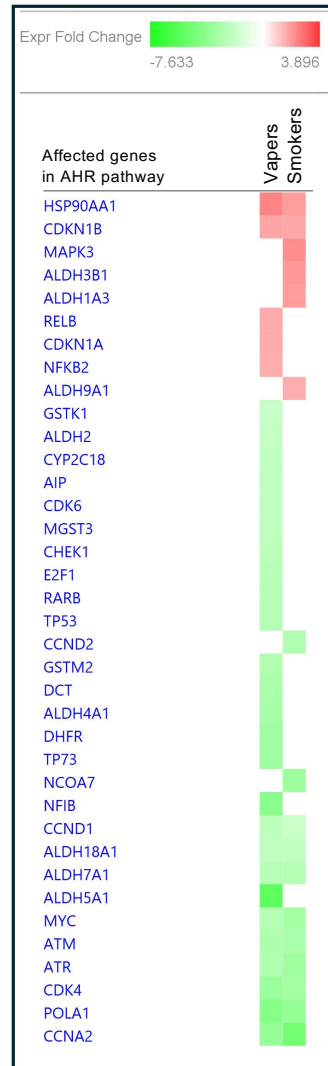

Fig. S4

Supplement: Supplementary file 4 [file DataSheet4.pdf]
