# Supplementary material for: Multidimensional exposure architecture shapes vaping-associated transcriptomic dysregulation in oral epithelium
Source: Front Oncol. 2026 Jun 1;16:1838256. doi: 10.3389/fonc.2026.1838256 (PMC13265642; doi:10.3389/fonc.2026.1838256)
Supplement: Supplementary file 5 [file DataSheet5.docx]

Supplementary Material

# Supplementary Data

**Supplementary Data 1.** Lists of differentially expressed genes (DEGs) identified in the primary and sensitivity models.

# Supplementary Figures and Tables

## Supplementary Figures

**Supplementary Figure 1.** Visualization of the intersections of the differentially expressed genes (DEGs) identified in various sensitivity models.

**Supplementary Figure 2.** Pairwise Spearman correlation matrices of exposure variables in vapers (Panel A) and smokers (Panel B).

**Supplementary Figure 3.** Comparative analysis of the AHR canonical pathway in vapers and smokers by IPA. The Comparison Analysis in IPA was used to identify trends or similarities and differences in canonical pathways across gene datasets. AHR signaling pathway analysis of differentially expressed genes in vapers (Panel A) and smokers (Panel B). Up-regulated and down-regulated genes are depicted as red and green nodes, respectively. Orange nodes, prediction of activation; blue nodes, prediction of inhibition. For clarity, only a section of the entire AHR signaling pathway is shown.

**Supplementary Figure 4.** Comparative analysis of DEGs belonging to the AHR pathway identified in vapers and smokers. Red squares: up-regulated genes; green squares: down-regulated genes.
